# Application of Binary Diagnostic Ratios of Polycyclic Aromatic Hydrocarbons for Identification of Tsunami 2004 Backwash Sediments in Khao Lak, Thailand

**DOI:** 10.1155/2014/485068

**Published:** 2014-07-22

**Authors:** Siwatt Pongpiachan

**Affiliations:** NIDA Center for Research & Development of Disaster Prevention & Management, School of Social and Environmental Development, National Institute of Development Administration (NIDA), 118 Moo3, Sereethai Road, Klong-Chan, Bangkapi, Bangkok 10240, Thailand

## Abstract

Identification of Tsunami deposits has long been a controversial issue among geologists. Although there are many identification criteria based on the sedimentary characteristics of unequivocal Tsunami deposits, the concept still remains ambiguous. Apart from relying on some conventional geological, sedimentological, and geoscientific records, geologists need some alternative “proxies” to identify the existence of Tsunami backwash in core sediments. Polycyclic aromatic hydrocarbons (PAHs) are a class of very stable organic molecules, which can usually be presented as complex mixtures of several hundred congeners; one can assume that the “Tsunami backwash deposits” possess different fingerprints of PAHs apart from those of “typical marine sediments.” In this study, three-dimensional plots of PAH binary ratios successfully identify the Tsunami backwash deposits in comparison with those of global marine sediments. The applications of binary ratios of PAHs coupled with HCA are the basis for developing site-specific Tsunami deposit identification criteria that can be applied in paleotsunami deposits investigations.

## 1. Introduction

Polycyclic aromatic hydrocarbons (PAHs), usually acknowledged as a group of persistent organic pollutants (POPs), have been comprehensively investigated in the past decades because these congeners have a profound association with a wide range of adverse health effects and other respiratory diseases [1–3]. PAHs are widely detected in various types of environmental compartments including marine organisms [4–9]. It is well known that PAHs can be generated from both anthropogenic and natural sources [10–13]. According to recent studies, particulate PAHs are harmful to human health due to their responsibilities for cancer, endocrine disruption, and reproductive and developmental effects [14–17]. As a consequence of distress over its potential hazard to public health, numerous studies were conducted to investigate the impact of meteorological parameters on its temporal variation and spatial distribution [18, 19]. Further attempts on clarification of factors governing diurnal variation of PAHs have also been carried out in different countries [20–23].

PAHs and other semivolatile organic compounds (SVOCs) have also been applied as chemical tracers to discriminate marine deposits from terrigenous components [24–28]. In particular, binary diagnostic ratios of PAHs can be employed to categorize anthropogenic and biogenic sources in marine deposits [28–30]. The molecular diagnostic binary ratio method for PAH source identification involves comparing ratios between pairs of frequently found PAH compound characteristics of different sources. Stationary source combustion emissions from the use of coal, oil, and wood are low in Cor (coronene) relative to B[a]P, while mobile source combustion emissions from diesel and petroleum use are high in B[g,h,i]P and Cor relative to B[a]P [31]. The ratio of these PAHs can be used to distinguish between traffic dominated PAH profiles and other sources [32, 33]. The ratio of certain PAH species (diagnostic binary ratio) can provide some information about the impact of different sources of PAHs in ambient air [34, 35]. Fl/(Fl + Pyr), B[e]P/(B[e]P + B[a]P), B[b,j,k]F/B[g,h,i]P, Ind/B[g,h,i]P, B[a]P/B[e]P, B[a]A/Chry, B[a]P/B[g,h,i]P, and Ind/(Ind + B[g,h,i]P) can be used as characteristic diagnostic parameters to identify their emission sources [30, 36]. For instance, Fl/(Fl + Pyr) and Ind/(Ind + B[g,h,i]P) ratios were used as indicators to discriminate fossil fuel from other modern biomass combustions, with low ratios (<0.40 and <0.20, resp.) signifying petroleum, intermediate ratios (0.40–0.50 and 0.20–0.50) indicating liquid fossil fuel (vehicle and crude oil) combustion whereas ratios higher than 0.50 can be considered as signals of wood or coal combustion [30].

Recently, Tipmanee et al. [37] used PAHs as “chemical proxy” to conduct source apportionment by using PCA technique in the Tsunami 2004 affected coastal area in the southern part of Thailand. They concluded that road dust and oil burning are two major sources of PAHs detected in marine surface sediments, indicating the importance of Tsunami backwash of terrestrial soils for coastal environments. However, in order to strengthen this conclusion, further investigations need to be performed. Without comparing the “fingerprint” of particulate PAH source profiles from various emission sources with those of marine deposits, it seems difficult to draw a conclusion by only relying on source apportionment technique. PCA offers the advantages of not requiring prior knowledge of the chemical composition and size distribution of emissions from specific sources (source profiles) but has the drawback of being mathematically indeterminate, allowing a wide range of possible solutions even when it is applied to relatively simple simulated data sets. The most difficult part of interpreting PCA is the explanation of negative correlations, which usually can be explained as contrast (when a parameter is growing in value the “negatively correlated one” is lowering its values). PCA has been applied to hundreds of environmental data matrices in order to interpret complex data structures on chemical pollution. Moreover, there are factorization techniques close to PCA that avoid this possible source of ambiguity as nonnegative matrix factorization, such as UNMIX and PMF. UNMIX is a multivariate receptor modeling package that inputs observations of particulate composition and seeks to find the number, composition, and contributions of the contributing sources or source types. This model also produces estimates of the uncertainties in the source compositions. UNMIX uses a generalization of the self-modeling curve resolution method developed by Henry, 1997 [38]. A major difference between UNMIX and PMF is that UNMIX does not make explicit use of errors or uncertainties in the ambient concentrations. This is not to imply that the UNMIX approach regards data uncertainty as unimportant, but rather that the UNMIX model results implicitly incorporate error in the ambient data.

In contrast to multivariate techniques such as PCA, visual comparison of PAH fingerprints and diagnostic binary ratios are preferable since they require no environmental insights of interpreting correlation coefficients of certain variables in each principal component and thus overcome the limitations of PCA. Overall, the main purposes of this study are to comprehensively investigate the fingerprints and diagnostic binary ratios of PAH aerosols from various emission sources in Songkhla province with marine deposits in Tsunami 2004 affected coastal area of Thailand. These findings will open a new window in applying PAHs as a “chemical proxy” to identify Tsunami backwash deposits and thus enhance the knowledge of Tsunami impacts on surficial sediment distribution in Khao Lak coastal area of the Andaman Sea.

## 2. Materials and Methods

### 2.1. Sampling Stations

#### 2.1.1. Sediments from Marine Environment

The research area is governed by the northeast monsoon from mid-October until March and the southwest monsoon from May to September and the intermonsoon phases. This study was carried out offshore along the west coast of Phang Nga province, Thailand, which was heavily affected by the 2004 Tsunami [39, 40]. The research area covers approximately 1,000 km^2^ between Thap Lamu and Pakarang Cape. The water depth of the surveyed area reached from 5 m down to 70 m. The sediment is built up by grain sizes from mud to gravel with a sediment thickness decreasing towards offshore (see Figures  S1–S3 available online at http://dx.doi.org/10.1155/2014/485068). Details of the research area are described in Feldens et al. [41].

#### 2.1.2. PM_10_ Monitoring Stations at Hat-Yai City

The first observatory station, Site-1, was placed at Novotel Centara Hat-Yai Hotel (7°00′20.65′′N 100°28′15.65′′E) at 30 m above the building basement and influenced mainly by transported pollution from traffic jams in the city center. Site-3 was positioned at Lee Gardens Grand Plaza Hotel (7°00′21.39′′N 100°28′15.94′′E) at 125 m above the ground level. Site-2, which was situated at Lee Gardens Grand Plaza Hotel at 60 m above the ground level, appears to be affected by a mixture of air masses including vehicular emissions, long-range transportation of aged particles and maritime aerosols. Intensive monitoring campaigns were conducted at all observatory stations simultaneously from December 17 to 20, 2007, in the relatively cold period. PM_10_ samples were collected every three hours continuously from 21:00 h December 17 to 21:00 h December 20 by using Graseby-Andersen.

#### 2.1.3. Sampling Site Descriptions of Various Emission Sources at Songkhla Province

Population of Songkhla Province is about 1.32 million occupying an area of approximately 7,394 km^2^. Songkhla is located 950 km south of Bangkok, situated on the eastern side of the Malayan Peninsula, bordering on Nakhon Sri Thammarat and Phatthalung to the north; Yala, Pattani, and States of Kedah (Sai Buri) and Perlis of Malaysia to the south; the Gulf of Thailand to the east; and Satun and Phatthalung to the west (Figure  S4). Hat-Yai, a district of Songkhla, is better known than the provincial capital itself as an economic and tourism zone of Songkhla and thus many industrial factories and stores are located in this area. Sampling site descriptions are given in detail as follows.

*Prince of Songkhla University (PSU)*. The site was situated at about 3 m above ground level at the Faculty of Environmental Management of Prince of Songkhla University and about 550 m away from the main traffic road that leads to the city center of Hat-Yai. It is important to note that PSU1 and PSU2 represent the sampling period of June (28–30 June, 2007) and October (24–26 October, 2007), respectively. This site is considered as an urban residential zone.

*Traffic Intersection (TI).* The station was located at the traffic intersection in front of the main gate of PSU. It is situated on the eastern side and approximately 2.5 km far away from the Hat-Yai city center. This station is regarded as a traffic area close to urban residential zone. The air samples were collected on 5–7 July, 2007.

*Corpse Incinerator (CI).* This station is a part of Kor-Hong temple, located at the northern side and about 1.5 km far away from TI. Since timbers and tires were generally used as fuel for corpse incineration, this site is considered as an emission source of both timbers and tires-burning. This site represents the sampling period of 19–21 July 2007. 

*Charoen Pokphand Factory (CPF).* This site was situated inside the fish can manufacturing factory of Charoen Pokphand group, which is the largest business conglomerate in Thailand. As crude oil was used for the fish can production, this station can be regarded as an emission source of crude oil burning. The monitoring was conducted on 24–26 July 2007. 

*Songkhla Lake (SL).* This station was located at the south of Songkhla Lake and approximately 13 km far away from the northern side of PSU. This site is also situated about 14 km away from the western side of the Gulf of Thailand. Since there are not many industrial and/or traffic emission sources including chemical and metallurgy factories and power plants in this district, we consider this site as a rural background sampling station. SL1 and SL2 represent the monitoring period of July (27–29 July, 2007) and October (20–22 October, 2007), respectively. 

*Rubber Sheet Manufacturing Factory 1 (RMF1).* This monitoring site was located at Tumbol Tungwan, Hat-Yai district. As a part of the manufacturing process, the rubber sheet was treated with steam of high temperature and high pressure coupled with the purification by using sulfuric acid solution. Since Para rubber trees were used as fuel for this process, this site represents an emission of mixed Para rubber trees burning, latex fragments, and sulfuric acid aerosols. The air samples were collected from 30 July to 1 August 2007. 

*Rubber Sheet Manufacturing Factory 2 (RMF2).* This station was situated at Tumbol Tachang, Banglum district. Both RMF1 and RMF2 are regarded as an emission of mixed Para rubber trees burning, latex fragments, and sulfuric acid aerosols. The air samples were collected from 2 to 4 August 2007. 

*Bus Terminal (BT).* This site was located at the south-western side of PSU and approximately 1.4 km away from the campus. This station was selected as a source of diesel emission because the majority of these buses are diesel-fueled. The air sample collection was started from 5 to 7 August 2007. 

*Waste Incinerator (WI).* This site was situated at the city center and belongs to the municipality of Hat-Yai city. Since the municipal waste incinerated is a heterogeneous mixture of solid wastes and burning fuels, this site can be recognized as a combination of solid waste burning and diesel exhaust emission. The air samples were collected from 28 to 30 August, 2007. 

*Barbeque Festival (BF).* This site was located inside the PSU campus on the top roof of Faculty of Natural Resources. The barbeque festival has become an annual tradition that is held on the second week in August. The 40th Annual Barbecue Festival is set for Wednesday, August 15, 2007. This site can be considered as an emission of charcoal burning. The air samples were collected from 15 to 18 August, 2007. 

*Phetkasem Road (PR).* This station was located at the heart of Hat-Yai city. The air mass collected at this area reflected the heaviest burden from traffic congestions with the mixture of diesel and benzene exhaust emissions. The monitoring was conducted on 27–29 August 2007. 

*Kor-Hong Hill (KHH).* This site was situated at the radio station on the top of Kor-Hong hill with the elevation of 356 meters. The air mass passed over the station was considered as a mixture of all emission sources in urban area and thus can be regarded as an urban residential zone monitoring site. The sampling was conducted on 3–5 November 2007.

*Rice Straw Burning (RSB).* The rice straw burning has been the major practice for removing rice straw because it is fast, economical, and practical in removing disease organisms. Although the options for the disposition of rice straw are limited, this practice leads to unacceptable air pollution. The station was situated at rice field in Satingpra district, Songkhla Province, and considered as a representation of biomass burning. The sampling was conducted on 16 November 2007. 

*Biomass Burning (BB).* As a part of soil preparation process, the biomass must be disposed of in order to make way for the plantation. The sampling site was adjacent to the unused land and located at Namom district, Songkhla Province. This site can be regarded as an emission source of biomass burning. The sampling was conducted on 17 November 2007.

*Para Rubber Tree Burning (PTB).* This station is located in Namom district, Songkhla Province, and can be recognized as an emission source of Para rubber tree burning. The air samples were collected on 18 November 2007.

### 2.2. Sampling Equipment

#### 2.2.1. Filter Sample Collection and Meteorological Data

In this study, Graseby-Anderson high volume air sampler (PM10-TE6001) was used to collect PM_10_ samples every 3 h consecutively with the flow rate of 1.132 m^3^ min^−1^. To avoid any contaminations, tweezers and aluminum foils were cleaned by dichloromethane (DCM) prior to use. All quartz fiber filters (47 mm Whatman quartz microfibre filters (QM/A)) were weighed gravimetrically on a microbalance Mettler Toledo AB204-S (Columbus, Ohio, USA) before and after sampling to quantify PM_10_ mass load. It is also worth mentioning that all filters were precleaned by DCM using Soxhlet extraction for 8 h prior to use to avoid any potential contamination. During the intensive monitoring campaign, all filters were kept in refrigerator at 4°C to minimize the loss of PAHs during sample preservation. Detailed description of sample collection has been published in Pongpiachan [42] and Pongpiachan et al. [43, 44].

#### 2.2.2. Hydroacoustic Equipment and Sediment Sampling

During the research cruises in November-December 2007 with RV CHAKRATONG TONGYAI and November-December 2008 with RV BOONLERT PASOOK, approximately 1500 nautical miles of hydroacoustic profiles (side scan sonar, multibeam echo sounder, and shallow reflection seismic with a boomer system) were recorded offshore Pakarang Cape. Based on these data sediment distribution maps were compiled. The grab samples discussed in this study were taken with a Van-Veen-type grab sampler, which was used to collect 70 surface sediment samples during 1–8 December 2007. Sediment samples were wrapped in clean aluminum foil, placed in a glass bottle, and kept frozen at −20°C. They were freeze-dried prior to being grounded and sieved to homogenize the samples and then kept in the refrigerator at −4°C until analysis.

### 2.3. PAHs Analysis

All organic solvents (i.e., DCM and Hexane) are of HPLC grade and are purchased from Fisher Scientific. A cocktail of 15 PAHs as determined by Norwegian Standard (NS 9815: S-4008-100-T) (phenanthrene (Phe), anthracene (An), fluoranthene (Fluo), pyrene (Pyr), 11 h-benzo[a]fluorene (11H-B[a]F), 11 h-benzo[b]fluorene (11H-B[b]F), benz[a]anthracene (B[a]A), chrysene (Chry), benzo[b]fluoranthene (B[b]F), benzo[k]fluoranthene (B[k]F), benzo[a]pyrene (B[a]P), benzo[e]pyrene (B[e]P), indeno[1,2,3-cd]pyrene (Ind), dibenz[a,h]anthracene (D[a,h]A), benzo[g,h,i]perylene (B[g,h,i]P), each 100 *μ*g mL^−1^ in toluene: unit: 1 × 1 mL) and a mix of recovery internal standard PAHs (*d*_12_-perylene (*d*_12_-Per), *d*_10_-fluorene (*d*_10_-Fl), each 100 *μ*g mL^−1^ in xylene: unit: 1 × 1 mL) were supplied by Chiron AS (Stiklestadveien 1, N-7041 Trondheim, Norway). Standard stock solutions of 4 *μ*g mL^−1^ of deuterated PAHs (used as internal standard) and 100 *μ*g mL^−1^ of native PAHs were prepared in nonane. Working solutions were obtained by appropriate dilution in n-cyclohexane. All solutions were stored in amber colored vials at −20°C. Silica gel (0.040–0.063 mm) was purchased from Merck. All materials used (silica gel, glass, and cotton wool, etc.) were Soxhlet-extracted with DCM for 24 h and kept dry (in desiccator) until use. The fractionation/cleanup and blow-down process followed the method reported by Gogou et al. [45]. The samples were analyzed for PAHs using Varian GC/MS-MS system comprising a CP-3900 gas chromatograph (Walnut Creek, CA, USA) with a 1077 universal injector and a three-dimensional quadrupole ion-trap selected ion storage mass spectrometer (Varian Saturn 2200). The target compounds were separated on a 60 m length × 0.25 mm i.d. capillary column coated with a 0.25 *μ*m film thickness. The chromatographic conditions coupled with the quantification and identification of PAHs were described in Pongpiachan et al. [43, 44]. Analysis of the congeners is based upon the principle of internal standard (IS) method. One of the essential requests of employing an IS is that it represents comparable physiochemical properties or the same type of substitution as the target compound. A relative response factor (RRF) for individual native congener was first analyzed. This is used for quantification, as the relative response between the IS and the native analyte should remain constant. It is an appropriate technique due to its recovery losses of the congener during extraction and analysis is expected to equal those of the IS. The computation of RRF can be explained as follows:

(1) $$\begin{array}{c} \text{RRF}=\frac{A_{\text{nat}}}{A_{\text{is}}}\times \frac{C_{\text{is}}}{C_{\text{nat}}}, \end{array}$$

where *A*_nat_ = peak area of the native compound in the standard; *C*_nat_ = concentration of the native compound in the standard; *A*_is_ = peak area of internal standard; *C*_is_ = concentration of the internal standard. The RRF_STD_ used for quantifying samples are the mean of those calculated for the two quantification standards run on the same day. Concentration (*C*) of analytes in sample extracts is calculated using the following formula:

(2) $$\begin{array}{c} C=\frac{A_{\text{nat}}}{A_{\text{is}}}\times \frac{1}{{\text{RRF}}_{\text{STD}}}\times \frac{W_{s}}{W_{\text{is}}}, \end{array}$$

where *W*_is_ = weight of IS added to the sample and *W*_s_ = weight or volume of the sample analyzed. Where *W*_is_ = weight of IS added to the sample, *W*_*s*_ = weight or volume of the sample analysed. A recovery determination standard (RDS) was used for the calculation of both internal standard (IS) and the sampling efficiency standard (SES) of recoveries during sample preparation and extraction/purification. A known amount of RDS was added at the final stage prior to GC/MS analysis and was assumed to suffer zero loss:

(3) %Recovery=[(AisARDS)S×(ARDSAis)STD] ×[(CisCRDS)STD×(CRDSCis)S]×100%,

where *A*_RDS_ is area of recovery determination standard and *C*_RDS_ is concentration of the recovery determination standard. Recoveries of IS were used as an indication of the analyte losses during extraction, preconcentration, cleanup/fractionation, and blow-down stages. The calculation of the sampling efficiency by using the sampling efficiency standard (SES) is described as follows:

(4) %Recovery  of  SES=[(AisASES)STD×(ASESAis)S] ×[(CisCSES)S×(CSESCis)STD]×100%,

where *A*_SES_ = area of the sampling efficiency standard and *C*_SES_ = concentration of the sampling efficiency standard. Recoveries of SES were used as an indication of analyte losses during sampling as opposed to the analysis. Analytical precisions and accuracies were calculated using the standard SRM 1941b. Mean recovery (based on extraction of matrix-matched certified reference materials (*n* = 8)) was in range of 77–119%. The precision of the procedure, calculated as relative standard deviation on the duplicate samples, was less than 15%. All sample concentrations were calculated using standardized relative response factors run with each batch [43, 44].

### 2.4. The Representativity of Diagnostic Binary PAHs Ratios

Standard deviations of a data set comprised of triplicate PM_10_ bound PAH samples (*n* = 3) from 15 different sources at Songkhla province were employed to evaluate the representativity of collected atmospheric PAH patterns. The average percentage standard deviation of binary ratios (i.e., average of Fluo/Pyr, B[a]A/Chry, B[a]P/B[e]P, and Ind/B[g,h,i]P) was considerably low ranging from 9.30% to 39.1% with the average of 18.9%. As a consequence, it appears reasonable to assume that the diagnostic binary ratios can be applied for the representativity of PM_10_ bound PAHs profiles collected from various emission sources.

## 3. Results and Discussion

### 3.1. Levels and Distribution Patterns of PAHs in Khao Lak Marine Sediments

**Figure 1:**
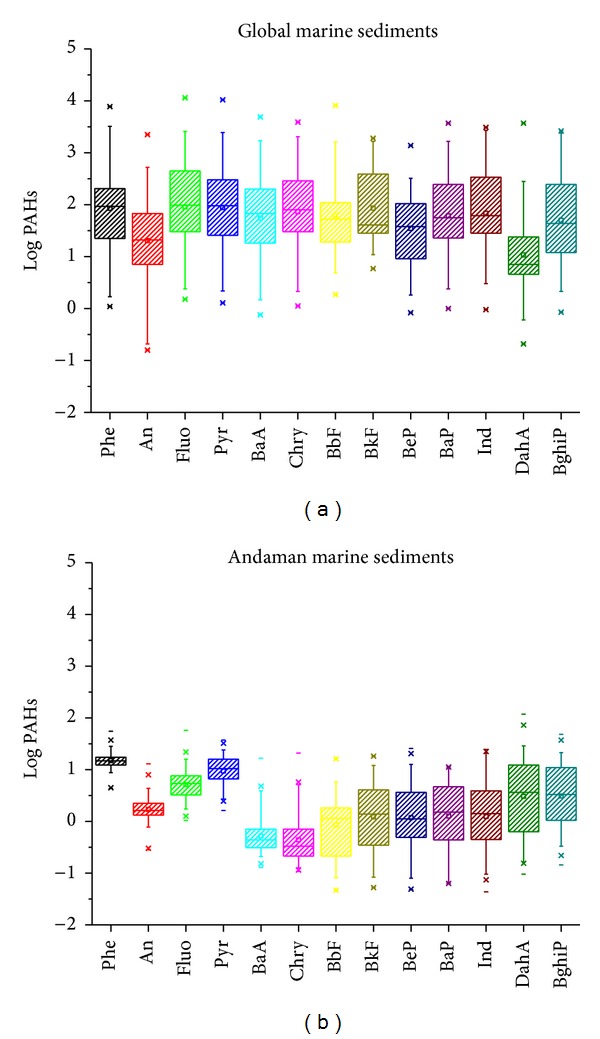
Box plots of PAHs in global marine and Andaman Sea sediments.

**Figure 2:**
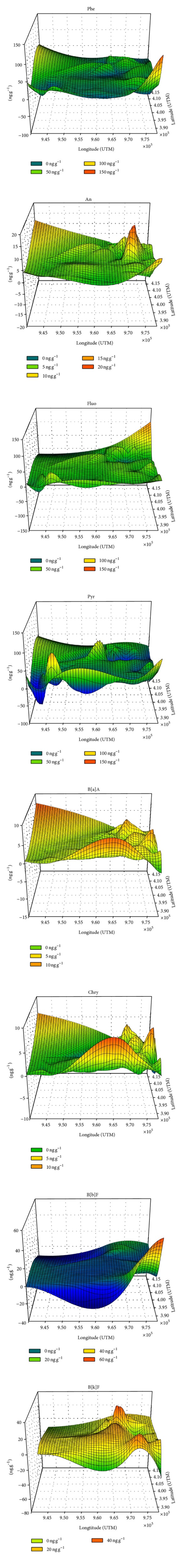
Distribution patterns of PAHs at the Tsunami affected coastal areas of Andaman Sea.

**Figure 3:**
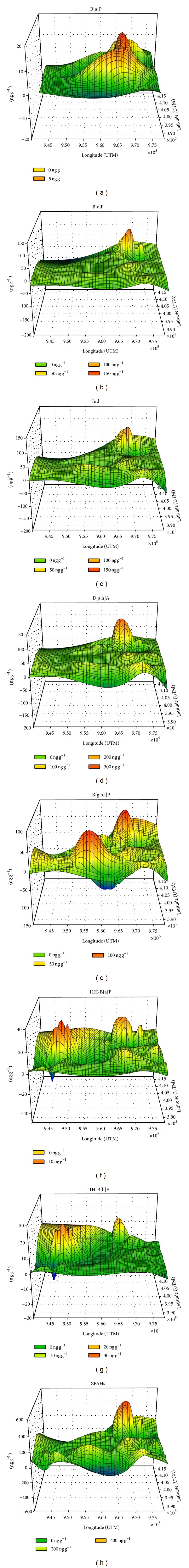
Distribution pattern of PAHs at the Tsunami affected coastal areas of Andaman Sea.

Table 1 and Figure 1 list the average concentration, minimum, maximum, and standard deviation of PAHs in the marine sediments of Khao Lak coastal area in comparison with those of other marine sediments around the world. The total concentrations of fifteen probably carcinogenic PAHs ranged from 12.58 ng g^−1^ to 278.10 ng g^−1^ with an arithmetic mean of 69.43 ± 70.67 ng g^−1^ as written in Table 2 and illustrated in Figures 2 and 3. In this study, the ∑_13_PAHs refers to the sum of analyzed Phe, An, Fluo, Pyr, B[a]A, Chry, B[b]F, B[k]F, B[a]P, B[e]P, Ind, D[a,h]A, and B[g,h,i]P. The observed ∑_13_PAHs were much lower than those values reported in harbor sediment of Boston (54,253 ng g^−1^), coastal sediments of Barcelona Harbor (15,069 ng g^−1^), riverine sediment of Guangzhou Channel (12,525 ng g^−1^), mangrove sediment of Hong Kong (3,714 ng g^−1^), coastal sediment of Cotonou (1,189 ng g^−1^), and coastal sediment of Carteau (210 ng g^−1^), but still higher than those values of coastal sediments in Rosas Bay (12 ng g^−1^), Santa Ponsa Bay (26 ng g^−1^), and Le Panier (34 ng g^−1^) [46–52].

### 3.2. Diagnostic Binary Ratios of PAHs

As illustrated in Table 1, the diagnostic binary ratios of Ind/(Ind + B[g,h,i]P), B[a]A/Chry, B[a]P/B[g,h,i]P, Fluo/(Fluo + Pyr), B[k]F/Ind, and An/(Phe + An) of Khao Lak coastal sediments were calculated and compared with previous studies of PAH fingerprints from various emission sources. Since the mean value of B[a]P/B[g,h,i]P (0.41 ± 0.70) was in the range of 0.3–0.78, it denoted the impact of oil burnings on study sites [53]. This interpretation is further supported by the results of B[a]A/Chry (0.96 ± 1.91), Fluo/(Fluo + Pyr) (0.35 ± 0.41), and An/(Phe + An) (0.11 ± 0.11), highlighting the influence of oil combustions over Khao Lak sediment samples (see Table 2 [28, 53–56]). Obviously, these binary diagnostic ratios are quite useful and provide valuable information to identify the potential sources of PAHs. However, these diagnostic ratios alone, as well as source profiles, should be used with great caution as physiochemical processes such as UV-photolysis and heterogeneous reaction with trace gaseous species can alter PAH distribution pattern during their transport from the emission source to the receptor site. Furthermore, several studies report the biodegradation of PAHs in sediments, particularly occurring under aerobic, sulfate reducing, and denitrifying conditions [57–59]. The stabilities of diagnostic binary PAH ratios are questionable, especially in aquatic environment surrounded by PAH-degrading bacteria [59]. In order to minimize the above-mentioned uncertainties, the plots of three- dimensional diagnostic ratios can enhance the reliability of binary ratios and thus be used as a tool to characterize sediment samples based on its PAH emission profiles.

**Figure 4:**
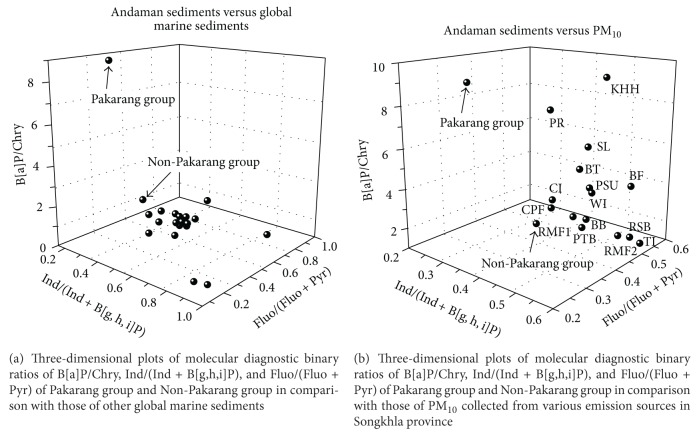
Diagnostic binary ratios of PAHs in Andaman Sea sediments and various types of PM_10_ collected in Songkhla province, Thailand.

Six PAH congeners have been selected and categorized into three-dimensional plots of molecular diagnostic binary ratios of Fluo/(Fluo + Pyr), Ind/(Ind + B[g,h,i]P), and B[a]P/Chry, which represents *x*-axis, *y*-axis, and *z*-axis, respectively. The clearest features in all categories (see Figures 4(a) and 4(b)) are as follows: (i) three-dimensional (3D) plots of Non-Pakarang group and other global marine sediments were grouped together (see Figure 4(a)); (ii) there are very clear different sources in Pakarang group, plausibly indicating the effect of Tsunami backwash (see Figure 4(a)); (iii) 3D plots of Non-Pakarang group were located in similar positions as those of RMF, CPF, CI, PTB, and BB suggesting that PAHs were conveyed from emission sources in Songkhla province to Khao Lak coastal area, then consequently to sediments (see Figure 4(b)); and (iv) 3D plots of Pakarang group highly deviate from the majority of plot members in both figures (see Figures 4(a) and 4(b)), which can be explained by the deposition of terrestrial components triggered by Tsunami backwash in Pakarang area.

Further attempts were examined to investigate the reliability of binary ratios in order to characterize the origins of sediment samples. Statistical descriptions of six binary ratios were illustrated in Table 2. There are significant differences between average values of An/(An + Phe), B[a]A/(B[a]A + Chry), B[a]P/(B[a]P + B[e]P), Ind/(Ind + B[g,h,i]P), and B[k]F/Ind in PM_10_ collected at Hat-Yai city and Khao Lak sediments. This can be explained by several reasons. Firstly, it can be postulated that urban aerosols from Hat-Yai city play a minor role in governing PAH distributions in Khao Lak sediments. Secondly, meteorological conditions can also alter particulate PAH profiles through UV-photolysis in fly ash particles [61, 62], aqueous phase [63], and heterogeneous reactions with ozone during the transport [64].

Thirdly, several studies report the importance of marine organisms for bioaccumulation of PAH compounds from dredged sediments [46, 65–67]. Hence, the differences of molecular diagnostic binary ratios of PAHs between Hat-Yai PM_10_ and Khao Lak sediments can be described as a consequence of bioaccumulation activities caused by marine organisms. Despite its varieties of emission sources, there are only three significant differences of average values of Fluo/(Fluo + Pyr), Ind/(Ind + B[g,h,i]P), and B[k]F/Ind observed in PM_10_ collected from 17 various emission sources and Khao Lak sediments. This finding reflects the fact that PAH contents in Khao Lak sediments were affected by more complicated emission sources rather than a single dominant point source.

### 3.3. Hierarchical Cluster Analysis (HCA)

**Figure 5:**
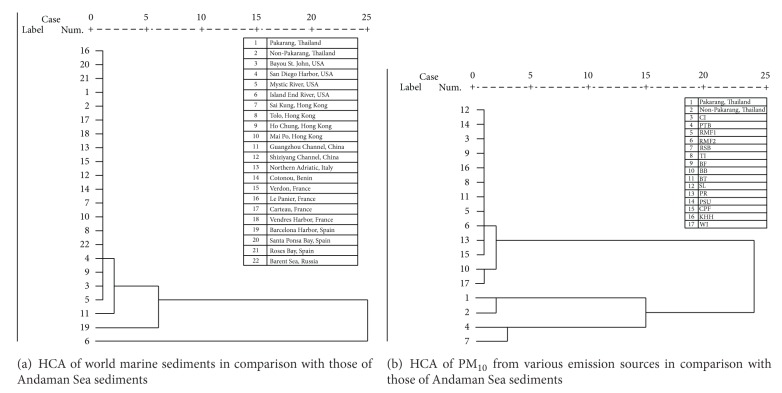
Hierarchical dendrogram for PAHs using average linkage between groups.

Cluster analysis (CA) seeks to identify homogeneous subgroups of cases in a population which both minimize within-group variation and maximize between-group variation. In this study, CA was conducted using SPSS 13.0 for Windows through Ward linkage on the correlation coefficient distance. Through the application of three diagnostic binary ratios, it seems reasonable to represent original PAHs data set in three dimensions and thus visualize key information that was hidden in the table, while working with only B[a]A/Chry, Fluo/(Fluo + Pyr), and Ind/(Ind + B[g,h,i]P) of the original data (see Figure 4). Cluster analysis can also assist in visualizing the data in two dimensions. It is also worth mentioning that HCA uses 100% of information available, whilst the applications of diagnostic binary ratios tend to rely on certain PAH congeners. In Figure 5, HCA of the PAH variables extracted from marine sediments and PM_10_ were examined through Ward linkage on the correlation coefficient distance.  Figure 5(a) discloses a single dominant group, which comprises 90% of HCA members, highlighting that the distribution pattern of PAHs in Khao Lak sediments is similar to other world marine sediments to some degree. Apparently with no surprise, both “Pakarang group” and “Non-Pakarang group” are clustered next to each other and grouped with sediments collected from Spain and France (i.e., Figure 5(a), no. 17, no. 18, no. 21, and no. 22). For instance, “Non-Pakarang group” is grouped next to “Carteau, France (i.e., Figure 5(a), no. 17),” a highly nutrient-rich site located in the Gulf of Fos and subjected to chemical pollution from industrial and petrochemical complex at Fos [68]. Furthermore, most of the PAH contaminations observed in Santa Ponsa Bay (Figure 5(a), no. 20) and Rosas Bay (Figure 5(a), no. 21) are of pyrolytic origin [46]. This interpretation is in agreement with those of PCA results conducted by Tipmanee et al. [37], which conclude that 61.4% of PAHs are of pyrolytic origin (i.e., road dust). In Figure 5(a), “no. 19 (i.e., Barcelona Harbor, Spain)” is clearly a “*runt,*” at least in the single linkage dendrogram, because no. 19 does not join the main group until the last step of clusterizing process. This can be explained by another dominant source of PAHs in this coastal area. By plotting the ratios of ΣMP/P (sum of the methyl-phenanthrene concentrations/phenanthrene concentration)* versus* the isometric ratio of Fluo/Pyr for all the mussels sampled in the Mediterranean Sea, Baumard et al. [46] discovered an overimposition of petrogenic PAHs in Barcelona Harbor marine sediments. As a consequence, the convincing dissimilarity of no. 19 sample observed in dendrogram of Figure 5(a) can be attributed to the strong influence of either oil spill or petroleum leakage in Barcelona Harbor sediments.

Figure 5(b) demonstrates the HCA dendrogram of PAHs observed in Khao Lak marine sediments and PM_10_ collected from 17 various emission sources in Songkhla province. This analysis reveals three large differentiated clusters. One of these clusters groups together several of the particulate PAH emission sources in Songkhla province. In other two clusters appear the remaining variables. Within these clusters, other groupings appear such as those formed by “Pakarang group (no. 1)” and “Non-Pakarang group (no. 2)” observed in the second cluster. The third cluster reveals two members of PTB (no. 4) and RSB (no. 7), which are grouped next to “Pakarang group” and “Non-Pakarang group.” Since PTB and RSB stand for “Para rubber tree burning” and “rice straw burning,” respectively, these findings reflect the possible influence of biomass burnings on distribution of PAHs in Khao Lak sediments. It is also worth mentioning that no. 1 and no. 2 are close to no. 17 (WI) and no. 10 (BB), which are clusterized in the first cluster of dendrogram (see Figure 5(b)). Since WI and BB stand for “waste incinerator” and “biomass burning,” one can assume some conceivable impacts of biomass burnings on PAH concentrations of Khao Lak coastal sediments in the Andaman Sea.

## 4. Conclusions

The Indian Ocean Tsunami of 2004 was only overshadowed by the 2011 Great East Japan Earthquake and Tsunami with more than 15,000 fatal casualties, but also the nuclear accidents and meltdowns in the Fukushima Daiichi Nuclear Power Plant. Apart from investigating significant concerns about the effect of PAH aerosols on human health, the comprehensive investigation of the “fingerprints” of PAH aerosols from marine deposits in 2004 Tsunami affected coastal areas of Thailand may contribute to the science leading to better Tsunami prediction.

Molecular diagnostic binary ratios of PAHs in Tsunami 2004 affected coastal sediments, Hat-Yai urban aerosols, and PM_10_ from various sources in Songkhla province were comprehensively investigated and compared. Three-dimensional plots of molecular diagnostic binary ratios successfully discriminate “Pakarang group” from other global marine sediment samples. Since hydroacoustic profiles show the impact of terrestrial deposits adjacent to “Pakarang Cape” coastal area [69, 70], it appears reasonable to ascribe Tsunami backwash 2004 to high deviations of “Pakarang group” sediment samples observed in three-dimensional plots of binary ratios. Interestingly, five of six binary ratios were significantly different when comparing the average values of PAH contents in PM_10_ collected at Hat-Yai city with Khao Lak sediments. This indicates that Khao Lak sediments have been influenced by more complicated emission sources rather than occupied by a long-range transportation of urban aerosols from Hat-Yai city. The application of HCA using PAH contents highlights the contribution of both pyrolytic combustions and biomass burning aerosols to distribution of PAH contents in Khao Lak coastal sediments. Overall, these findings may open a new window in using binary PAH ratios to investigate the paleotsunami from core sediments in other coastal regions and thus deeper insights into the science of Tsunami.

## Supplementary Material

Supplementary materials include the information of maps of PM_10_ observatory sites and sampling locations of marine sediment samples as well as multi-beam echo sounder data and grab samples obtained in November and December 2007 during the cruise with RV CHAKRATONG TONGYAI.

## Figures and Tables

**Table 1:** Diagnostic binary ratios of PAHs, together with average, standard deviation, minimum and maximum of PAH concentrations (ng g^−1^ dry weight) in marine sediments collected at the study sites.

	Number of rings	Molecular formula	Molecular mass	Average (*n* =70)	Standard deviation	Minimum	Maximum	Percentage contribution
Phe	3	C_14_H_10_	178	15.77	6.19	4.33	37.17	22.71
An	3	C_14_H_10_	178	1.94	1.26	0.28	7.92	2.79
11H-B[a]F	4	C_17_H_12_	216	1.40	2.50	N.D.	12.82	2.02
11H-B[b]F	4	C_17_H_12_	216	0.82	1.31	N.D.	9.62	1.18
Fluo	4	C_16_H_10_	202	6.19	4.32	1.04	21.86	8.92
Pyr	4	C_16_H_10_	202	11.46	7.26	1.63	37.58	16.51
B[a]A	4	C_18_H_12_	228	0.69	0.86	0.13	4.84	0.99
Chry	4	C_18_H_12_	228	0.72	1.12	0.11	5.76	1.04
B[b]F	5	C_20_H_12_	252	2.10	3.28	0.05	16.06	3.02
B[k]F	5	C_20_H_12_	252	3.17	4.12	0.05	18.10	4.57
B[e]P	5	C_20_H_12_	252	2.93	4.33	0.05	20.55	4.22
B[a]P	5	C_20_H_12_	252	2.66	3.08	0.06	11.15	3.83
Ind	6	C_22_H_12_	276	3.30	5.21	0.04	22.32	4.75
D[a,h]A	5	C_22_H_14_	278	9.86	17.93	0.10	117.46	14.20
B[g,h,i]P	6	C_22_H_12_	276	6.42	7.90	0.15	48.02	9.25
∑PAH				**69.43**	**70.67**	**12.58**	**278.10**	**100.00**

Ind/(Ind + B[g,h,i]P)	Diesel: 0.35–0.70 [55]			0.34	0.87			
B[a]A/Chry	Coal: 1.0–1.2 [53], gasoline: 0.28–1.2 [53, 55]			0.96	1.91			
B[a]P/B[g,h,i]P	Coal: 0.9–6.6, vehicles: 0.3–0.78, oil burning: >2 [53]			0.41	0.7			
Fluo/(Fluo + Pyr)	Coal: 0.53 [55], gasoline: 0.40 [56]			0.35	0.41			
B[k]F/Ind	Diesel: 0.5 [60], wood combustion: 0.6 [60]			0.96	1.96			
An/(Phe + An)	Petroleum: <0.1, combustion: >0.1 [28, 54]			0.11	0.11			
B[a]P/∑PAH-_5rings_*				0.13	0.45			

*∑PAH-_5rings_ is the sum of B[a]P, D[a,h]A, B[e]P, B[b]F, and B[k]F.

**Table 2:** Diagnostic binary ratios of PAHs in Andaman marine sediments in comparison with those of particulate PAHs from other emission sources.

	Andaman (*n* = 70)	Hat-Yai (*n* = 72)	Emission sources (*n* = 17)	*t*-test	*t*-test
				Andaman versus Hat-Yai	Andaman versus emission sources
An/(An + Phe)	0.11 ± 0.11	1.16 ± 0.134	0.13 ± 0.11	S∗	NS∗∗
Fluo/(Fluo + Pyr)	0.35 ± 0.41	0.72 ± 0.120	0.50 ± 0.053	NS	S
B[a]A/(B[a]A + Chry)	0.96 ± 1.91	1.16 ± 0.053	0.51 ± 0.098	S	NS
B[a]P/(B[a]P + B[e]P)	0.48 ± 1.05	0.88 ± 0.13	0.41 ± 0.15	S	NS
Ind/(Ind + B[g,h,i]P)	0.34 ± 0.87	0.46 ± 0.13	0.46 ± 0.074	S	S
B[k]F/Ind	0.96 ± 1.96	0.68 ± 0.13	0.22 ± 0.16	S	S

*S: significant (*P* < 0.05), ∗∗NS.
