# Supplementary material for: Application of Binary Diagnostic Ratios of Polycyclic Aromatic Hydrocarbons for Identification of Tsunami 2004 Backwash Sediments in Khao Lak, Thailand
Source: ScientificWorldJournal. 2014 Jul 22;2014:485068. doi: 10.1155/2014/485068 (PMC4130332; doi:10.1155/2014/485068)
Supplement: Supplementary file 1 — Supplementary materials include the information of maps of PM10 observatory sites and sampling locations of marine sediment samples as well as multi-beam echo sounder data and grab samples obtained in November and December 2007 during the cruise with RV CHAKRATONG TONGYAI. [file 485068.f1.pdf]

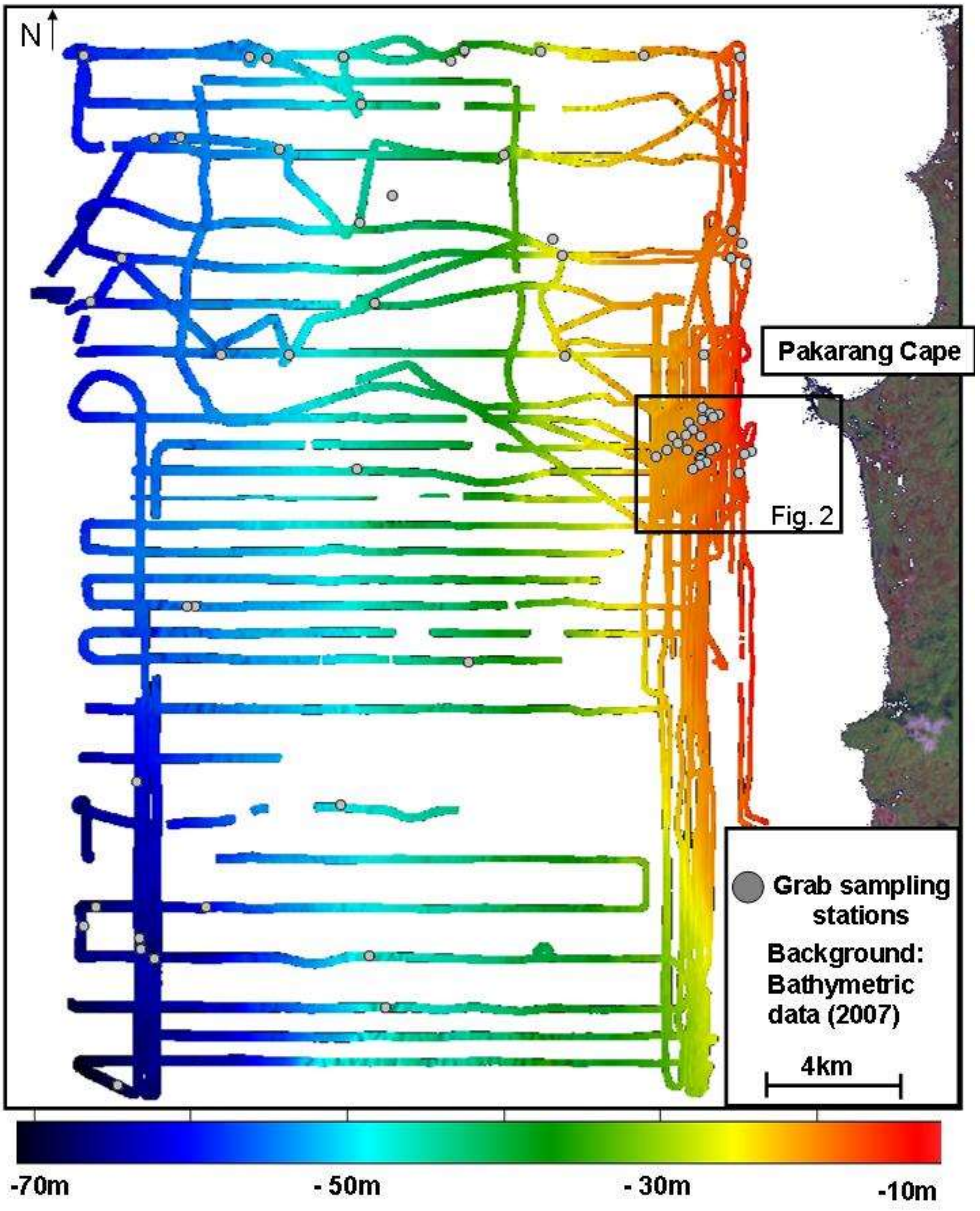

Fig. S1. Overview of multibeam echo sounder data and grab samples obtained during the 2007 cruise with RV CHAKRATONG TONGYAI. Bathymetry based on multibeam echo sounder data. Grab samples were taken during a cruise with RV CHAKRATONG TONGYAI in November and December 2007.

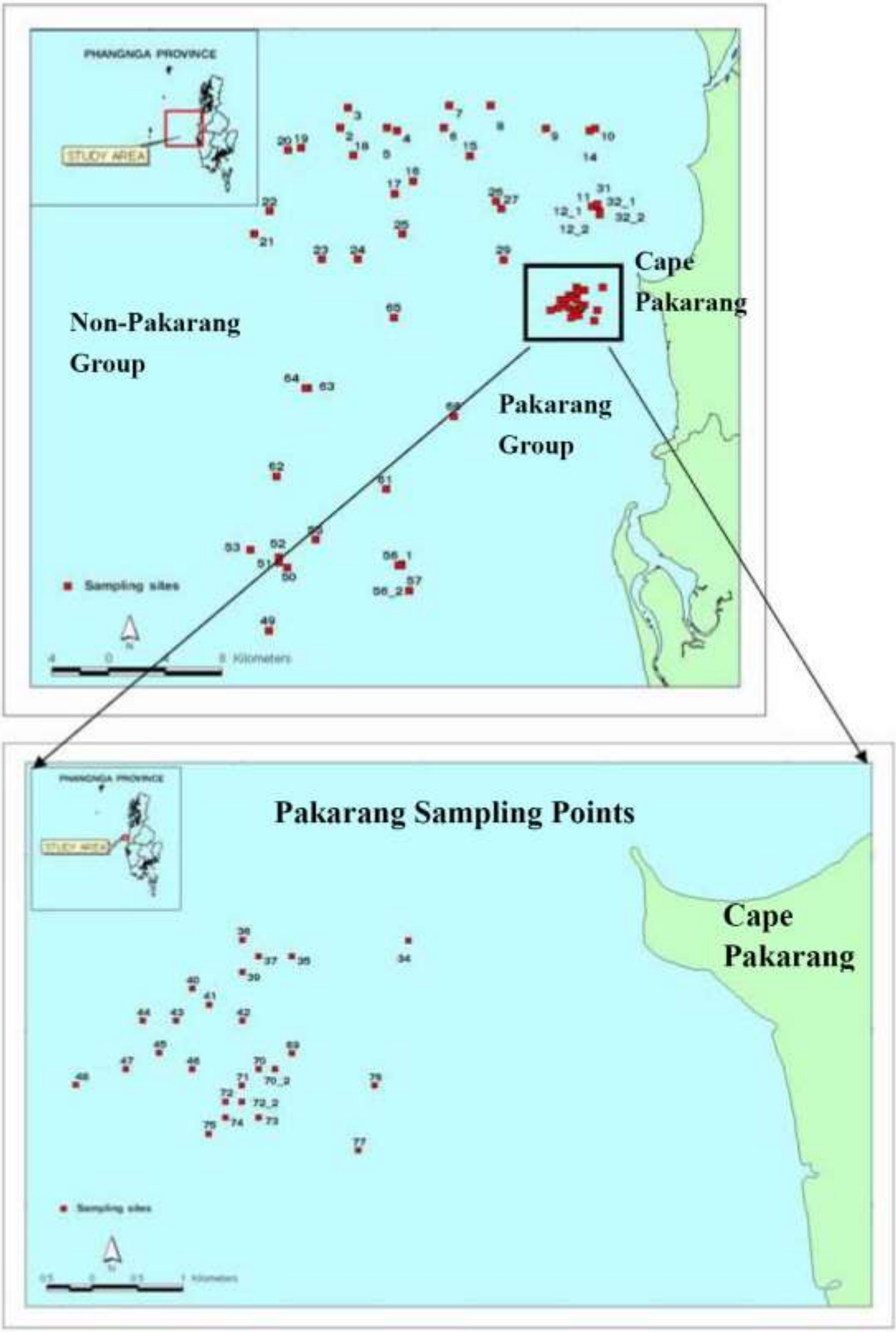

Fig. S2. Sampling site locations at the tsunami-affected coastal areas of Andaman Sea.

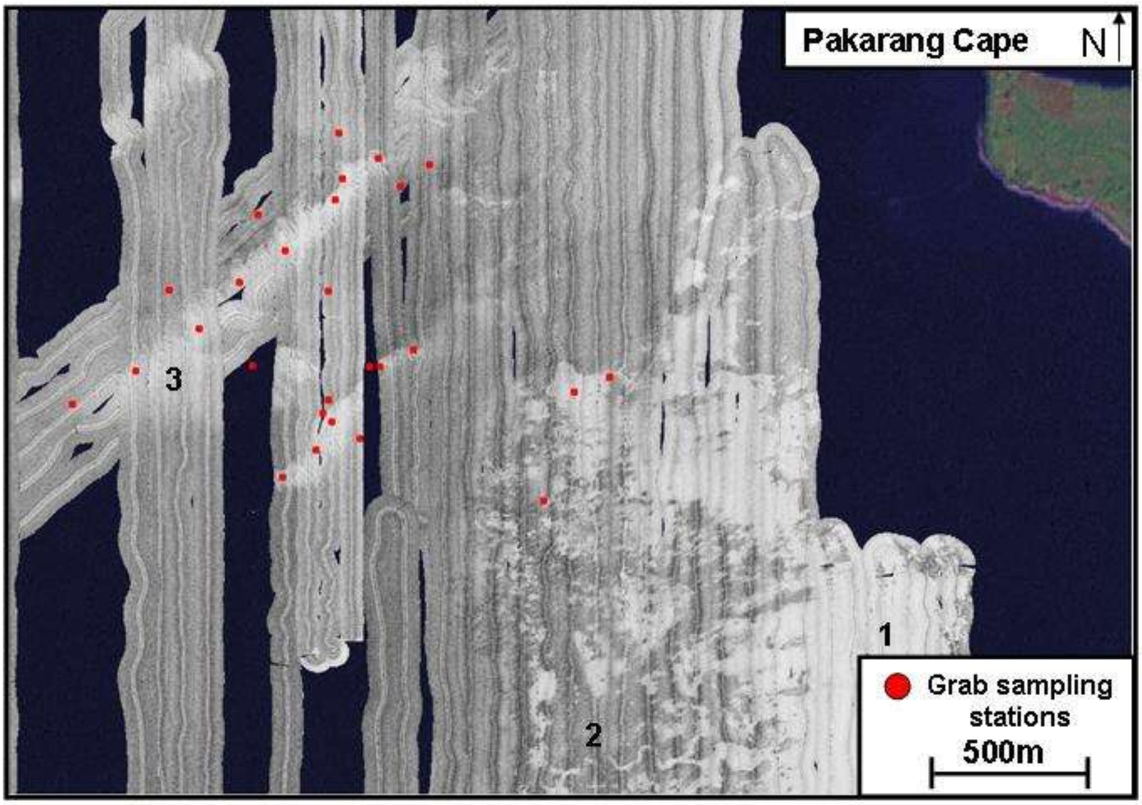

Fig. S3. Side Scan Sonar data (taken during the 2008 cruise with RV BOONLERT PASOOK) around Pakarang Cape. 1: Fine-grained sediments occur close to the coastline, in water depths between 5 and 10 m. 2. One of several small scale channels (width between 25 and 60 m) apparent in the side scan sonar data. 3: Area of elevated sediment transport structures. Sediment distribution is based on side scan sonar mapping. White colours reflect fine grained sediment, dark colours reflects more coarse grained sediment (sand).

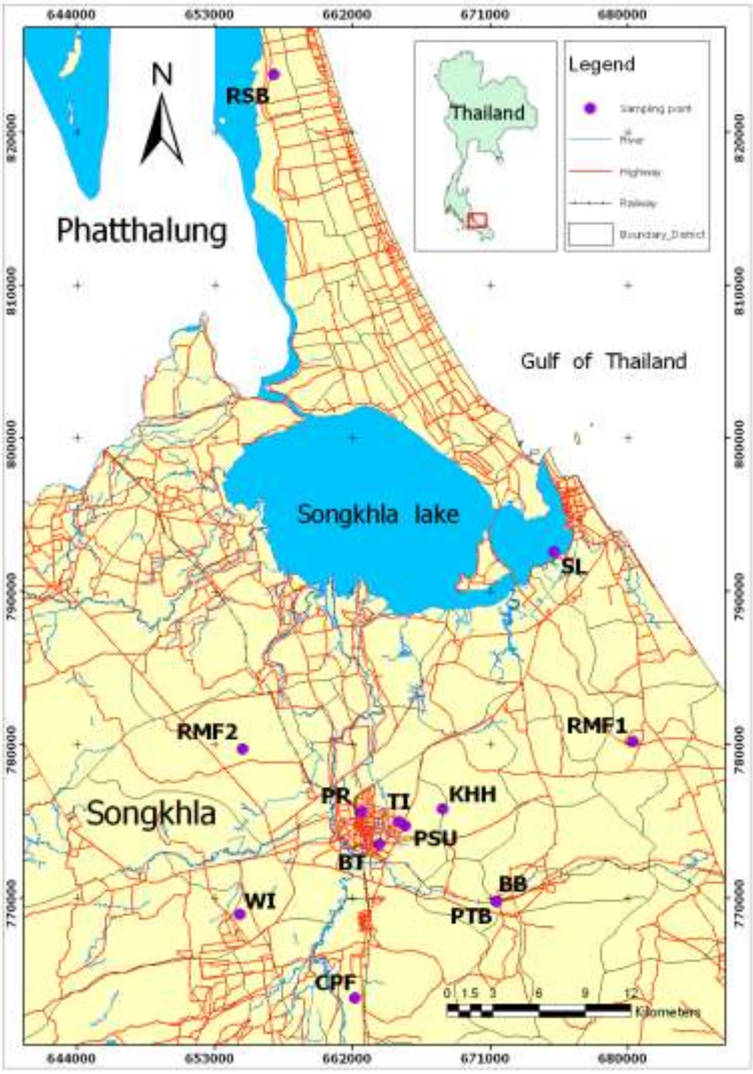

Fig. S4. Map of PM_{10} sampling sites.
